# Physiological Anti-Müllerian Hormone Concentrations in Male and Female Dogs and Cats before and around Puberty

**DOI:** 10.3390/ani14172561

**Published:** 2024-09-03

**Authors:** Tanja Alexander, Ulrike Flock, Ruth Klein, Sven Reese, Andrea Meyer-Lindenberg, Beate Walter

**Affiliations:** 1 Clinic of Small Animal Surgery and Reproduction, Centre for Clinical Veterinary Medicine, Faculty of Veterinary Medicine, Ludwig-Maximilians-University, Veterinaerstr. 13, 80539 Munich, Germanyandrea.meyer-lindenberg@lmu.de (A.M.-L.); 2 Laboklin GmbH & Co.KG, Steubenstr. 4, 97688 Bad Kissingen, Germany; r.klein@laboklin.com; 3 Chair of Anatomy, Histology and Embryology, Department of Veterinary Sciences, Faculty of Veterinary Medicine, Ludwig-Maximilians-University, Veterinaerstr. 13, 80539 Munich, Germany; sven.reese@lmu.de; 4 Small Animals Clinic, Centre for Clinical Veterinary Medicine, Faculty of Veterinary Medicine, Ludwig-Maximilians-University, Veterinaerstr. 13, 80539 Munich, Germany

**Keywords:** anti-Müllerian hormone, reproduction, dog, cat, bitch, queen, tomcat

## Abstract

**Simple Summary:**

The diagnostic value of anti-Müllerian hormone (AMH) is well established but less is known about physiological concentrations in young individuals. The aim of this study was to determine AMH levels in small animals before and around puberty and to correlate these levels with testosterone in males entering puberty. Blood samples were collected between 2 and 48 weeks after birth for AMH and testosterone measured using a chemiluminescence immune assay. AMH concentrations were higher in males than in females, with no significant differences among male individuals. However, queens had significantly higher AMH concentrations compared to bitches. A significant decreased in AMH was observed in the males beginning 24 weeks after birth, which correlated only in dogs with an increase in testosterone. In bitches, AMH remained below the detection limit until 16 weeks after birth after which it increased slightly. In queens, AMH was detectable from week 2 with a significant increase starting at week 8, even approaching the chemiluminescence immune assay’s upper limit. In conclusion, queens secreted AMH much earlier and in significantly higher amounts than bitches. The decrease of AMH in male species entering puberty was observed in both dogs and tomcats, but a negative correlation with testosterone was derived only for male dogs.

**Abstract:**

In recent years several studies established the diagnostic value of anti-Müllerian hormone (AMH) in companion animals. However, less is known about physiological AMH concentrations in young individuals highlighting the necessity to apply the diagnostic findings to this group. The aim of this study was to determine the AMH values of healthy male and female dogs between the age of 8 and 48 weeks, tomcats under 8 weeks and up to 48 weeks of age and queens between 2 to 12 weeks of age. In total, 96 blood samples were collected. Anti-Müllerian hormone was measured in all samples and testosterone was measured in the oldest age group of the males in both species. The hormones were analyzed using a human based chemiluminescence immune assay. Overall, AMH concentrations were higher in males than in females (*p* < 0.001). According to the AMH concentration there was no difference in males, but queens had significant higher AMH concentrations than bitches (*p* < 0.001). AMH remained high in males up to week 24 and decreased significantly thereafter (tomcats: *p* = 0.015; male dogs: *p* = 0.013), which correlated with an increase in testosterone levels for male dogs only. In bitches, AMH remained below the detection limit until the week 16 and slightly increased subsequently. In queens, AMH was detectable from the beginning with a significant increase in the older age group (*p* = 0.003). Half of the cats in the older age group even approached the chemiluminescence immune assay’s upper limit. The results show that female cats secrete AMH much earlier than female dogs in which AMH secretion begins just shortly before the start of the puberty. In the male animals, the decrease in AMH concentration around puberty was similar in dogs and cats, but a correlation with the increase of testosterone was only observed in dogs. Further research is required to determine the origin of the high AMH concentrations in female kittens and the lack of correlation between testosterone and AMH concentrations in male kittens.

## 1. Introduction

Anti-Müllerian hormone (AMH), a glycoprotein of the transforming growth factor-ß-family, is secreted primarily by Sertoli cells in males and granulosa cells in females. The key function of AMH is the regression of the Müllerian ducts during embryogenesis in males, in which high levels are secreted [1]. In females, under the absence of AMH, the Müllerian ducts progress into the outer epithelium of the ovaries, the salpinx, the uterus, the cervix, and the cranial part of the vagina during embryogenesis [2]. After puberty, AMH inhibits the recruitment of primordial follicles into the pool of growing follicles and reduces the responsiveness of growing follicles to the follicle stimulating hormone in females [3]. In males, AMH has a paracrine action on Leydig cells and reduces testosterone secretion [4]. Recent studies showed the expression of AMH and its receptor-2 in the prostate, lungs, brain, and hypophyses as well, but the specific action of AMH has not yet been determined [5].

Over the last decade, several studies have proven the diagnostic value of AMH in small animal reproduction. So far, AMH levels have helped to distinguish between intact and castrated dogs and cats [6,7,8], to identify bitches and queens with ovarian remnant syndrome [9,10,11], or to diagnose scrotal Sertoli cell tumor in male dogs and granulosa cell tumor in bitches [12,13]. Significantly higher AMH concentrations, in comparison to healthy animals, have been described in cryptorchid dogs and dogs with other disorders of sexual development [14,15] as well as in male dogs with deslorelin-induced testicular atrophy [16]. Furthermore, physiological factors influencing AMH concentration in dogs and cats can be observed during the estrous cycle and among different breeds and age groups. Significant changes in AMH levels over the estrous cycle in bitches have been reported with a rise in serum concentration of AMH from the transition of anestrus to proestrus and a significant decrease in AMH level from postovulatory estrus to metestrus in dogs [17,18]. In contrast, queens reached the highest AMH concentrations in anestrus and the lowest during heat [18,19]. Relating to the size, giant breed bitches had lower AMH values than medium and small size female dogs and the same authors described a gradual decline in AMH in adult dogs older than 4 years of age [20]. However, less is known about the AMH concentration in prepubertal cats and dogs. One study in cats has described measurable AMH serum concentrations in five female and male cats between 4 and 8 weeks after birth as AMH concentrations above 23 ng/mL. Further, it has been described that younger queens have higher AMH concentrations than adult cats [18,19,21,22]. In contrast, prepubertal bitches under 6 months of age seem to have low AMH concentrations [6,9,23,24]. To the authors’ knowledge, nothing is known about the AMH serum concentration in prepubertal male dogs, but a positive immunoreaction with AMH antibodies has been confirmed in the testes of fetal male dogs from the 36th day of pregnancy up to 48 days after birth [16,25,26].

The aim of this study was to investigate the time of the postnatal rise in AMH concentration in female dogs and cats and the postnatal decrease in AMH level in male dogs and cats, as well as its relation to the rise in testosterone as it is described in other species.

## 2. Materials and Methods

### 2.1. Animal and Blood Sample Collection

This study included 15 prepuberal bitches, 17 male dogs, 10 queens, and 21 tomcats. The animal population was drafted from Ludwig Maximilian University small animal clinic patients and shelter animals. This study was approved by the ethical commission of the veterinary faculty Ludwig Maximilian University Munich (no. 228-13-07-2020) and the owners gave their consent. The animals in this study were in general good health, proper body condition, and showed normal external genital organs. This study was designed cross-sectionally, and blood sample collections were performed once in 63 animals and twice in 33 animals during general health checks or antibody titer controls. The samples were grouped according to species, gender, and age of the animal, as shown in Table 1. Each group included 8 samples. The dogs consisted of 13 different breeds: mixed breed (*n* = 10), border collie (*n* = 1), Louisiana Catahoula leopard dog (*n* = 1), poodle (*n* = 4), Rhodesian ridgeback (*n* = 5), Pomeranian (*n* = 3), Maltese (*n* = 2), labrador (*n* = 1), Akita Inu (*n* = 1), cocker spaniel (*n* = 1), American bulldog (*n* = 1), Doberman (*n* = 1), and Deutsche Bracke (*n* = 1). The cats consisted of the following breeds: Bengal cats (*n*=15), Norwegian forest cats (*n* = 4), and European short-haired cats (*n* = 12). The weight distribution was between 1.00 kg and 27.50 kg (median: 12.25 kg) in the dog sample and between 0.38 kg and 4.40 kg (median: 2.40 kg) in the cat sample. In total, 96 blood samples were collected. Blood was obtained by puncture of the saphenous or cephalic vein with a 25-gauge needle. Blood samples were centrifuged at 600 G for ten minutes, and serum was drained directly afterwards and stored at −20 °C until the measurement of AMH. Testosterone was measured additionally in the oldest group of male dogs and tomcats to determine the phase of puberty. The female kittens were chosen before puberty and the bitches before their first heat.

### 2.2. Hormone Analysis

AMH was measured using a chemiluminescence immunoassay on a cobas E602 analyser (Roche) using murine anti-AMH antibodies validated for cats [18]. In dogs, the intra-assay co-efficient of variation was 1.8% and the inter-assay co-efficient of variation was 7.4%. In addition, the intra-assay co-efficient of variation was calculated for serial dilutions (1:2, 1:5: 1:10, 1:20) using physiological salt solution as a diluting agent. The intra-assay co-efficients of variation were 4.51% at 23.61 ng/mL, 2.52% at 1.30 ng/mL and 6.85% at 0.81 ng/mL. Comparing the expected values to the measured values after dilution in the dogs’ sample gave a recovery rate of 88.89% to 104.94%. Visual inspection of the data plotted in a scatter diagram and a correlation analysis (*r*² = 99.0%) confirmed the linearity under dilution. Recovery of human AMH standard added to canine plasma showed changes in optical density parallel to the AMH standard curve. Minimum detection limit of the AMH test was 0.01ng/mL, and maximum detection limit was 22.96 ng/mL. Testosterone concentrations were determined using a chemiluminescence immunoassay. Minimum detection limit of the testosterone test was 0.07 ng/mL and maximum detection limit was 15.00 ng/mL. The intra-assay co-efficient of variation in male dogs was 10.00% and in tomcats 10.98%, and the inter-assay co-efficient of variation in male dogs was 4.94% and in tomcats 5.21%.

### 2.3. Statistical Analysis

Statistical analysis was carried out by using SPSS 28.0.1.0 (IBM, Ehningen, Germany). AMH concentrations were displayed as mean ± standard deviation and median with range. Statistical analyses used unifactorial and multifactorial methods. Unifactorial statistical analysis for independent data employed Mann–Whitney U test, and Spearman’s Rho was used as a non-parametric test to measure the strength of association between two variables, where the value *r* = 1 means a perfect positive correlation and the value *r* = −1 means a perfect negative correlation.

AMH concentrations were compared between sexes for each species and between species for each sex using the Mann–Whitney U test. In addition, the dependence of the AMH concentration in males and females on age was determined.

The Spearman’s Rho method was used to determine the dependence of AMH concentration in males and females on age, and on testosterone levels in male animals over 20 weeks of age.

## 3. Results

The median, minimum, and maximum AMH concentrations within the different groups classified according to their species, gender, and age can be seen in Table 2. The AMH concentration was significantly higher in males than in females within each species (*p* < 0.001). There was no significant difference in the mean AMH concentration between the male cats and male dogs (*p* = 0.682). In contrast, the median AMH concentration in queens was significantly higher than in bitches (*p* < 0.001).

**Figure 1:**
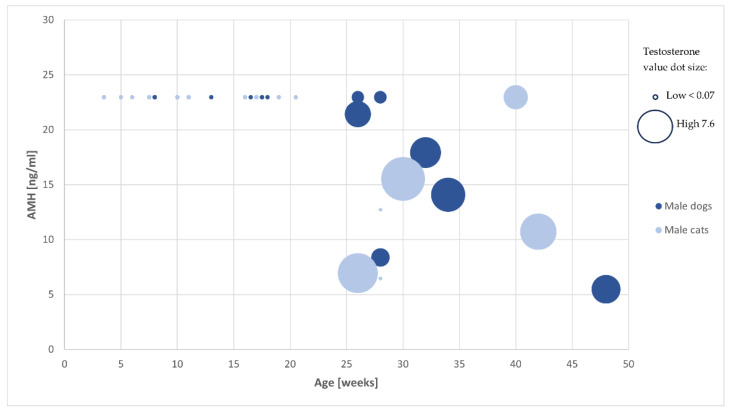
Dot plot of AMH values (*Y*-axis) in the examined tomcats (light blue) and male dogs (dark blue) in relation to their age (*X*-axis) showing the testosterone value by the size of the dots.

As displayed in Figure 1, the entire male dog and tomcat populations reached the chemiluminescence immune assay’s upper limit (>22.96 ng/mL) up to week 24 after birth and declined afterwards. Between week 24 and 48, there was a subsequent significant decrease in AMH serum concentration in tomcats (*p* = 0.015) and male dogs (*p* = 0.013). The AMH serum concentration decrease correlated negatively with the increasing testosterone concentration in male dogs; in tomcats, the increase in testosterone did not correlate with the decrease in AMH.

**Figure 2:**
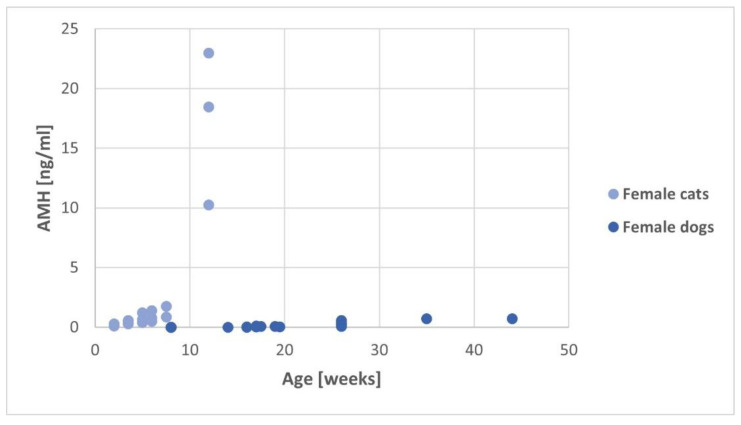
Dot plot of AMH values (*Y*-axis) in all examined queens and bitches in relation to their age (*X*-axis).

In queens and bitches, AMH concentration increased significantly with increasing age (queen: rho = 0.899, *p* < 0.001; bitch: rho = 0.931, *p* < 0.001), as shown in Figure 2. However, the AMH concentration increase starts earlier in queens and reaches significantly higher levels than in bitches (*p* < 0.001). The bitches had no detectable AMH levels up to week 16 (<0.01 ng/mL). Between week 16 and 24, the AMH concentration slowly increased in seven out of eight female dogs and every bitch older than 24 weeks had evident AMH values. In contrast, every queen in this study had measurable AMH concentrations, with a significant increase in AMH concentration in the older age group (*p* = 0.003), and half of the cats older than six weeks even reached chemiluminescence immune assay’s upper limit.

## 4. Discussion

The use of the AMH serum concentration as a diagnostic tool requires knowledge of the physiological secretion. The purpose of this study was to determine the physiological AMH serum concentrations in adolescent female and male dogs and cats and to identify any correlation between the increase in testosterone and decrease in AMH levels in male individuals around puberty, a subject which has barely been researched.

To the author’s best knowledge, this is the first study to examine AMH serum concentrations in correlation to age and testosterone concentration in male dogs and tomcats under 6 months. The AMH concentrations of all examined male dogs and tomcats in this study reached the chemiluminescence immune assay’s upper limit up to week 20. This matches the finding in one other study in which five tomcats between 4 and 8 weeks also had AMH concentrations above 23 ng/mL [19]. These high concentrations of AMH are most likely due to immature Sertoli cells that are present in the testes of prepubertal individuals, and which secret higher amounts of AMH than mature Sertoli cells [26,27,28]. In human male embryos, AMH secretion starts at the end of the fifth week of pregnancy, whereas in male embryos of dogs, it has been described from 35th day of pregnancy in [26,29]. After birth, the AMH concentration stays high in male youth until puberty [30]. During this time, the Sertoli cells grow and pass the premature development stage [29]. With the activation of the hypothalamic–pituitary–gonadal axis at the beginning of puberty, the secretion of androgens increases, which leads to maturation of the Sertoli cells and subsequently reduction in their growth rate and AMH secretion [31,32].

After week 20, a continuous decrease in AMH concentration was observed for male dogs and tomcats in this study. In male calves, an AMH peak with five months of age has been described [33] and in male kids, a temporary decrease in AMH concentration within the first week after birth followed by an increase in the second week has been described [34]. To mitigate a prepubertal peak of the AMH concentration in male dogs and tomcats, further studies with repeated measurements in the same individuals from birth until puberty and dilution of the serum samples are required.

A decrease in AMH concentration in correlation with an increase in testosterone concentration at the time of puberty has already been described in bulls in 1977 with a graded organ culture assay to detect AMH activity [35]. A decrease in AMH concentration in the oldest male individuals was also noted in this study and confirms the lower AMH concentrations described in post-pubertal male dogs and tomcats [7,8]. In the male dogs, the AMH concentration correlated negatively with the testosterone concentrations, as has also been described in horses, in which a negative correlation between AMH and testosterone in young stallions from puberty until adulthood has been determined [28,33]. However, there was no correlation between the increase in testosterone concentration and AMH concentration in the tomcats in this study. In two tomcats’ AMH decreased at a time where testosterone was still not measurable. A possible explanation could be a difference in the testosterone concentration in the blood serum and in the testicles, as described in men. In them, a negative correlation between the testosterone concentration in blood serum and in the testicles have been described [36]. In male lambs, the AMH concentrations in the testicular veins and the rete testis fluid have been determined, but the authors could not find a direct correlation [37]. Further studies are needed to examine if the decrease in AMH concentration in blood serum is correlated to an increase in testicular testosterone in tomcats and if there is an increase in the AMH concentration in seminal plasma around puberty in male dogs and cats.

On the other hand, there were two tomcats in this study with increasing testosterone concentrations and remaining high AMH concentrations. A possible explanation for this phenomenon could be that the decrease in AMH concentration took place at a higher level, and we were not able to notice it because the concentrations were above the upper detection limit of the test system. To examine this further a dilution of the samples would be necessary, but there was no serum left.

In male youths, it has been shown that the decrease in the AMH concentration around puberty is not only caused by reduced secretion of AMH, but also related to a change in the direction of its release [38]. It has been described that from puberty on, higher amounts of AMH are secreted into the spermatic tubules and less in the blood. Hence, the concentration of AMH in seminal plasma increases whereas in blood serum a decrease takes place [38]. In stallions, no correlation between the AMH concentration in blood serum and seminal plasma could be found [28]. Whether there are any correlations in male dogs and cats needs to be examined in further studies.

In female kids, the AMH concentration stays low until puberty and is sometimes not even measurable [39,40]. The highest AMH concentrations in female kids are reached on average by 15.8 years [41]. Afterwards, AMH stays high until the age of 25 years before it starts its continual decline [40]. In one study, prepubertal females had significantly lower AMH serum concentrations than males at the same age, but all females had measurable AMH concentrations [42].

In female dogs, the AMH concentration did not reach the chemiluminescence immune assay’s detection limit in the first 16 weeks in the current study. Between weeks 16 and 24, seven out of the eight female dogs had measurable but low AMH serum concentrations. This finding matches the result of another study in which a canine-specific AMH test was used and the authors determined measurable AMH concentration in prepubertal bitches between 4 and 6 months of age [9]. In another study in which a human-specific ELISA test comparable to the one used in the current study, only half of the female dogs between 12 and 24 weeks had AMH concentrations above the detection limit [6]. A possible explanation may be that AMH was not measurable in the youngest dogs examined, but the authors did not mention the specific age of the bitches in which AMH was determined.

Female calves with higher AMH concentrations between 8 and 16 weeks of age reached puberty earlier than individuals with lower AMH concentrations at the same age [43]. Further, the AMH concentrations at week 16 after birth correlated positively with the AMH concentrations 4 to 6 weeks after puberty. Thus, the authors postulated that AMH can be used as a fertility marker in calves at this young age, because of the interference of the AMH concentration with the antral follicle count. There were also differences in the rise in AMH concentrations in the female dogs in this study, but whether the AMH concentrations in prepubertal female dogs can be used to predict the start of the first heat—due to the described start of the AMH secretion in the ovaries with the development of epithelial cells of primordial follicles to granulosa cells of the primary follicles [3]—has to be investigated in further studies.

In contrast, the female kittens in this study already had AMH concentrations above the detection limit from week 2 with a subsequent rapid increase. In half of the queens, between weeks 8 and 12, AMH concentrations reached the chemiluminescence immune assay’s upper limit. A previous study has described significantly higher AMH concentrations in female kittens under three months of age than in adult queens [21]. Other studies also determined higher AMH concentrations in younger individuals between 4 and 12 months [18,19,22,44]. Later on, a decline in AMH concentrations seems to occur, because in adult cats, lower AMH concentrations with mean concentrations of 5.5 ng/mL, 7.3 ng/mL, and 5.65 ng/mL have been described [18,22,44]. A decline in the AMH concentration has also been described in women, bitches and mares, but in these species, it occurs later in the reproductive life [20,45,46]. The very high AMH concentrations in female kittens contrast with the AMH secretion patterns in other females in which the rise in AMH started later on [3,6,9,21]. In female dogs, the AMH concentration stayed low for 12 to 16 weeks after birth and increased only slightly afterwards, as mentioned above [6,9]. In female calves, it has been described that the AMH concentration stays constantly low over the first year of life [33], whereas in mares, a slight increase in the AMH concentration has been detected between 24 and 28 weeks after birth [47]. In female sheep lambs, a prepubertal peak in the AMH concentration has been noted [48], but the extremely high AMH concentrations in female kittens are unlikely a prepubertal peak because they were measured several months before the onset of puberty in this study and are still high even in young individuals [18,22]. Another possible explanation for the high AMH concentrations in queens may be cross reactions of other serum components with the ELISA test system [49,50]. However, in a previous study using the same test system, significant changes in AMH concentration over the estrous cycle within the same cats has been noted; therefore, cross-reactions cannot be the only reason for the overall high AMH serum concentrations in young cats, although they cannot be totally excluded [18]. Comparable high AMH serum concentration as in young female kittens has been determined in cats during anestrus. There is no plausible explanation for the origin of these high AMH values. At both times, there should not be much ovarian activity with numerous growing follicles, which are responsible for secreting the highest amounts of AMH in women and other females [47,51,52,53,54]. Further studies in female cats are needed to examine the ovaries of cats with high AMH serum concentrations to determine the origin of AMH. And studies in the future are needed to determine if there are breed-related differences, or other influences like environmental and also longitudinal studies in the same individuals will probably provide further insights in AMH secretion in these species.

## 5. Conclusions

Anti-Müllerian hormone concentrations were measurable in queens from early age on with a significant increase in the older age group in which half of the cats even exceed the chemiluminescence immune assay’s upper limit. Female cats secrete AMH much earlier than female dogs, in which the AMH secretion begins shortly before puberty with lower AMH concentrations. The decrease in AMH concentration in the male group entering puberty followed a similar pattern for both species, but a correlation with the increase in testosterone was only observed in the canines. Further research is required to determine the factors for the high AMH concentrations in female kittens and the lack of correlation between testosterone and AMH levels in tomcats.

## Figures and Tables

**Table 1:** Experimental group division by species, gender, and age.

Species	Gender	Age in Weeks and Size of Group					
Cats	Female	2 to 6 8 animals	-	6 to 12 8 animals	-	-	-
	Male	-	<8 8 animals	-	8 to 16 8 animals	16 to 24 8 animals	24 to 48 8 animals
Dogs	Female	-	-	-	8 to 16 8 animals	16 to 24 8 animals	24 to 48 8 animals
	Male	-	-	-	8 to 16 8 animals	16 to 24 8 animals	24 to 48 8 animals

**Table 2:** Median, minimum, and maximum AMH and testosterone concentrations within the different groups classified according to their species, gender, and age.

Species	Gender	Age [Weeks]	AMH [ng/mL]			Testosterone [ng/mL]		
			Median	Minimum	Maximum	Median	Minimum	Maximum
Cats	Female	2 to 6	0.4	0.12	1.22	-		
		6 to 12	20.71	0.87	22.96	-		
	Male	<8	22.96	22.96	22.96	-		
		8 to 16	22.96	22.96	22.96	-		
		16 to 24	22.96	22.96	22.96	-		
		24 to 48	14.12	6.46	22.96	5.5	<0.07	7.6
Dogs	Female	8 to 16	0.01	0.01	0.01	-		
		16 to 24	0.035	0.01	0.1	-		
		24 to 48	0.245	0.07	0.56	-		
	Male	8 to 16	22.96	22.96	22.96	-		
		16 to 24	22.96	22.96	22.96	-		
		24 to 48	19.68	5.49	22.96	1.95	0.3	4.4

## Data Availability

The original contributions presented in the study are included in the article, further inquiries can be directed to the corresponding author.
